# Synthesis, Biological Evaluation and Machine Learning Prediction Model for Fluorinated *Cinchona* Alkaloid-Based Derivatives as Cholinesterase Inhibitors

**DOI:** 10.3390/ph15101214

**Published:** 2022-09-30

**Authors:** Alma Ramić, Ana Matošević, Barbara Debanić, Ana Mikelić, Ines Primožič, Anita Bosak, Tomica Hrenar

**Affiliations:** 1 Department of Chemistry, Faculty of Science, University of Zagreb, Horvatovac 102A, 10000 Zagreb, Croatia; 2 Institute for Medical Research and Occupational Health, Ksaverska cesta 2, 10000 Zagreb, Croatia

**Keywords:** *Cinchona* alkaloid derivatives, cholinesterase inhibitors, multivariate linear regression models

## Abstract

A series of 46 *Cinchona* alkaloid derivatives that differ in positions of fluorine atom(s) in the molecule were synthesized and tested as human acetylcholinesterase (AChE) and butyrylcholinesterase (BChE) inhibitors. All tested compounds reversibly inhibited AChE and BChE in the nanomolar to micromolar range; for AChE, the determined enzyme-inhibitor dissociation constants (*K*_i_) ranged from 3.9–80 µM, and 0.075–19 µM for BChE. The most potent AChE inhibitor was *N*-(*para*-fluorobenzyl)cinchoninium bromide, while *N*-(*meta*-fluorobenzyl)cinchonidinium bromide was the most potent BChE inhibitor with *K*_i_ constant in the nanomolar range. Generally, compounds were non-selective or BChE selective cholinesterase inhibitors, where *N*-(*meta*-fluorobenzyl)cinchonidinium bromide was the most selective showing 533 times higher preference for BChE. In silico study revealed that twenty-six compounds should be able to cross the blood-brain barrier by passive transport. An extensive machine learning procedure was utilized for the creation of multivariate linear regression models of AChE and BChE inhibition. The best possible models with predicted R^2^ (CD-derivatives) of 0.9932 and R^2^(CN-derivatives) of 0.9879 were calculated and cross-validated. From these data, a smart guided search for new potential leads can be performed. These results pointed out that quaternary *Cinchona* alkaloids are the promising structural base for further development as selective BChE inhibitors which can be used in the central nervous system.

## 1. Introduction

The neurodegenerative disease generally implies a range of conditions that primarily affect the neurons in the human brain and cause problems with movement and/or mental functioning. The most common disease characterized by progressive non-reversible mental deterioration is Alzheimer’s disease (AD), which is caused mainly by a loss of cholinergic innervation in the cerebral cortex and characterized by decreased levels of the neurotransmitter acetylcholine in neurons [1,2]. This has led to the development of AD drugs that inhibit the activity of acetylcholine hydrolyzing enzymes, acetylcholinesterase (AChE), and butyrylcholinesterase (BChE). Today, four out of six currently in use drugs for the treatment of AD are AChE or non-selective cholinesterase inhibitors: donepezil, rivastigmine, galantamine, and pyridostigmine. All four drugs exhibit their pharmacological effect by increasing the amount of ACh in the brain consequently leading to temporally improved cognitive abilities of patients and alleviation of the AD symptoms. The problem in the development of AD drugs that can slow or stop the progression of disease lies in AD’s highly complex etiology associated with diverse clinical hallmarks which do not occur at the same time and whose intensities of occurrence vary which makes it difficult to establish a timely diagnosis. The diagnosis is usually made when the progression of AD is in an already advanced stage when symptoms of mental deterioration are pronounced. Besides inhibition of AChE, several different strategies have been used in the development of drugs able to target other mechanisms involved in the pathogenesis of AD-like amyloid-β (Aβ) peptide deposits, oxidative stress, dyshomeostasis of biometals, and hyperphosphorylated tau protein [3]. Although the effectiveness of ACh inhibition in the treatment of AD has been debated in recent years [4], after the discovery of AChE’s role in Aβ aggregation when was demonstrated that the AChE peripheral anionic site interacts with Aβ forming a stable AChE-Aβ complex which is more toxic than Aβ peptide aggregates [5,6], the search on AChE inhibitors was boosted again [7]. The focus was shifted to the search for dual binding site inhibitors able to interact with the catalytic anionic site and peripheral anionic site of AChE as a promising way of treating AD. Until recently, the main strategy in developing AD drugs was inhibition of AChE activity, but a few years back BChE also appeared in the focus of research since its activity with the progression of AD increased compared to the activity of AChE that significantly decreased with the time [8,9]. Since the treatment of AD and related dementias represents an increasing problem in global health and is becoming a more and more economic burden, especially on a global level, the development and optimization of new cholinesterase inhibitors with improved pharmacological properties than those in clinical use is an on-going hot research topic.

**Figure 1:**
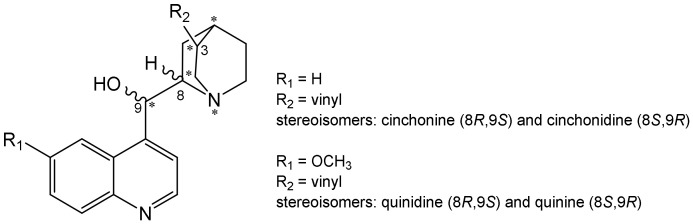
General structures of main *Cinchona* alkaloids. “*” denotes five chiral centers in the molecules.

Cinchonidine (**CD**) and cinchonine (**CN**) together with quinine and quinidine are the most known members of the *Cinchona* alkaloids family, Figure 1. Their structure consists of a rigid quinuclidine ring with the vinyl group, aromatic quinoline moiety, and hydroxymethyl unit connecting them. All compounds have five stereocenters but differ in only two of them, C8 and C9. However, since they behave like enantiomers in stereoselective reactions, they are called *pseudo-enantiomers*.

Because of their unique structure with a few available places for derivatization, *Cinchona* alkaloids and their derivatives are one of the most versatile groups of natural compounds [10]. Major applications of *Cinchona* alkaloid derivatives are in numerous organic reactions as chiral organocatalysts and chromatography for the separation of various racemic analytes as a chiral stationary phase Besides diverse chemical applications, they are also bioactive compounds. The most known bioactive alkaloid is quinine, which was used as a powerful antimalarial drug for centuries [11]. Derivatives bearing *Cinchona* scaffold were identified as autophagy inhibitors, potential antibiotics, anticancer or insecticidal agents [12,13,14,15].

In our previous study, we have identified several potent BChE inhibitors based on quaternary CD and CN scaffolds with high selectivity toward BChE [16]. Those results have encouraged us to continue research on *Cinchona* based alkaloids as potential cholinesterase inhibitors with selectivity toward BChE. We synthesized additional 46 CN and CD derivatives that differ in the structure of the quinuclidine quaternary nitrogen atom. In the benzyl moiety fluorine atom(s) were introduced to explore the influence on the compound’s bioactivity. Fluorine atoms are highly electronegative and relatively small in size, and they can be used in medicinal chemistry as bioisosteres of functional groups (e.g., hydrogen atom and methyl group), and serve as functional mimetics of the carbonyl group. Trifluoromethyl group can be bioisostere of *iso*-propyl and *tert*-butyl groups, replacing also the iodine or bromine atom. Furthermore, fluorine atoms and groups are important because of the ability to tune up more than one molecular property such as change of the conformation constraints, change of bioactivity of the molecule, membrane permeability (lipophilic properties), metabolic pathways, etc. Thus, derivatization of bioactive compounds with fluorine atoms is a well-known strategy in drug discovery [17,18]. Also, we have prepared a small series of corresponding 10,11-dihydroderivatives by reduction of the vinyl group in CD and CN to investigate whether this structural modification has influence on the inhibitory activity toward AChE and BChE. The *in vitro*-determined affinity of the studied compounds toward cholinesterases, their inhibition selectivity as well as the stereoselectivity of the enzymes, were analysed and defined by molecular modelling and extensive machine learning procedures. In addition, the ability of compounds to cross the blood-brain barrier by passive transport was evaluated as one of the desired features that CNS active compounds should have.

## 2. Results and Discussion

### 2.1. Synthesis

Since our pilot study on CD and CN derivatives of *Cinchona* alkaloids gave promising results regarding the inhibition potency of those compounds towards human cholinesterases [18], we have synthesized a series of 46 quaternary derivatives of Cinchona derivatives of which 18 were not previously described and/or mentioned in the chemical literature as stated in the experimental section.

Here we expanded our previous study on benzyl derivatives of CD’s and CN’s on those containing the EWG like fluorine atom, trifluoromethyl (CF_3_) or trifluoromethoxy groups (OCF_3_) on benzene. A small series of CD and CN compounds with the reduced vinyl group on quinoline ring was also synthesized. 10, 11-Dihydrocinhconidine (DHCD) and 10, 11-dihydrocinhconine (DHCN) were prepared by catalytic transfer hydrogenation of commercially available CD and CN. Quaternary derivatives of CD, CN, DHCD and DHCN were prepared by refluxing alkaloid (CD, CN, DHCD or DHCN) and appropriately substituted benzyl bromide in 2-propanol. Eighteen compounds: CD 2F-6CF_3_, CD 2F-4Br, CD 2Cl-6F, CN 2,3-F, CN 2,4-F, CN 2,6-F, CN 3OCF_3_, CN 2F-6CF_3_, CN 2F-4Br, CN 2Cl-6F, DHCN 3F, DHCD 4F, DHCN 4F, DHCD 3CF_3,_ DHCD 3OCF_3_, DHCN 3OCF_3_, DHCD 4OCF_3_ and DHCN 4OCF_3_ are new compounds. Other prepared compounds are already described in literature [19,20,21,22,23,24,25,26]. All compounds were prepared in moderate to good yields and characterized by standard analytical methods. Characteristic proton signals in the ^1^H NMR spectrum which can be used to distinguish between stereoisomers are signals of the vinyl group and methylene group of the benzyl moiety. ^1^H NMR spectra of 10,11-dihydro CD and CN lack signals of the vinyl group and instead have signals characteristic to the ethyl group. Their quaternary derivatives have signals in ^1^H NMR spectra with similar chemical shifts as unmodified DHCD and DHCN.

### 2.2. Inhibition of Cholinesterases

**Figure 2:**
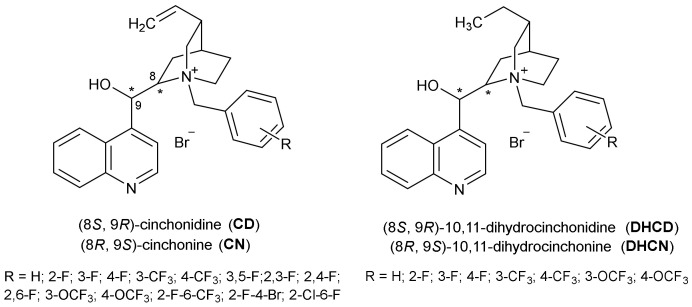
Structures of prepared compounds: CD Bzl (R=H); CD 2F; CD 3F; CD 4F; CD 3CF_3_; CD 4CF_3_; CD 3,5F; CD 3,4F; CD 2,3F; CD 2,4F; CD 2,6F; CD 3OCF_3_; CD 4OCF_3_; CD 2F-6CF_3_; CD 4Br-2F; CD 2Cl-6F; DHCD; DHCD Bzl (R=H); DHCD 3F; DHCD 4F; DHCD 3CF_3_; DHCD 4CF_3_; DHCD 3OCF_3_; DHCD 4OCF_3_; CN Bzl (R=H); CN 2F; CN 3F; CN 4F; CN 3CF_3_; CN 4CF_3_; CN 3,5F; CN 3,4F; CN 2,3F; CN 2,4F; CN 2,6F; CN 3OCF_3_; CN 4OCF_3_; CN 2F-6CF3; CN 4Br-2F; CN 2Cl-6F; DHCN; DHCN Bzl (R=H); DHCN 3F; DHCN 4F; DHCN 3CF_3_; DHCN 4CF_3_; DHCN 3OCF_3_; DHCN 4OCF_3_. “*” denotes two chiral centers at positions 8 and 9 in *pseudo-enantiomers*.

In this paper we broadened our previous research on the binding affinity of CD’s and CN’s derivatives toward BChE and AChE [18] and all synthesized compounds were tested as human BChE and AChE inhibitors. Since the effect of fluorine carrying groups on the binding affinity of the parent compound is difficult to predict, we synthesized a series of fluorine-containing substituents on benzyl group of CD’s and CN’s to enable us to carry out structure-activity relation studies concerning the inhibition of human cholinesterases. In line with that, we synthesized a series of fluorine and fluorine-containing CD’s and CN’s where the position of fluorine atom(s) and fluorine-containing groups was changed systematically (Figure 2). Moreover, according to our previous study [18], bioactive conformers of the *Cinchona* derivatives had different positioning of the vinyl group in the AChE and BChE active sites and are positioned to allow favorable orientation of other moieties in the molecule.

All synthesized compounds reversibly inhibited BChE and AchE, and as a measure of their inhibition potency the dissociation constants (± standard errors) of the enzyme-inhibitor complex (*K*_i_) were determined. The impact of the introduction of fluorine atoms in the structure of inhibitors and the impact of reduction of the vinyl group on inhibition potency were analyzed.

#### 2.2.1. Inhibition by Cinchonine and Cinchonidine Derivatives

The impact of changes in substituents on the benzene ring on inhibition potency can be analyzed separately for CD’s and CN’s. The *K*_i_ constants (± standard errors) for BChE and AChE are presented in Table 1.

The activity of BChE was inhibited by all tested compounds with *K*_i_ constants ranging from 0.075–9.9 µM. The most potent inhibitors were CD 3F and CD 3,5F, both with *K*_i_ constants around 76 nM which classified them as highly potent BChE inhibitors [27] and suggested the possible importance of *meta*-positioned fluorine atom (position 3) on inhibition potency. This is additionally supported by the fact that the *ortho* (CD 2F) and *para* (CD 4F) monofluorine substituted compounds didn’t reach such high potency of inhibition, being up to 19 times less potent inhibitors. Substitution of *meta*-fluorine with other electron-withdrawing groups-trifluoromethyl group in CD 3CF_3_ or trifluoromethoxy group (OCF_3_) in CD 3OCF_3_ led to the decrease of inhibition potency in order CD 3F > CD 3OCF_3_ > CD 3CF_3_ implying that inhibition potency of compounds with a substituent in *meta* position on benzene ring depend also on its volume due to the steric constraints of enzyme’s active site. In the case of CF_3_ or OCF_3_ as substituents, inhibition potency was similar whether they were in *meta* or *para* position. Five compounds with two substituents were synthesized as well, and from the values of their *K*_i_ constants it seems that for achieving optimal interactions with BChE active site, *meta* positions are preferred. The most potent disubstituted inhibitor is the one with fluorine atoms on positions 3 and 5. The introduction of other halogen or CF_3_ beside *ortho* positioned fluorine atom, revealed that *para* position for additional halogen is preferred, e.g., CD 2F–4Br. Inhibition potency of CD 3F and CD 3,5F is comparable with one determined for CD’s with unsubstituted benzyl moiety, and benzyl moiety with *meta*-positioned methyl group as determined previously [18]. Moreover, if the inhibition potential of compounds studied in this work was compared to CD’s with other *meta*-positioned substituents on benzyl moiety, it can be concluded that for achieving optimal position and binding interactions, halogens larger than fluorine are not desirable.

Contrary to CD’s, different positions of fluorine atom alone or in combination with additional fluorine atoms, fluorine carrying groups or other halogens have minor or no effect on the inhibition potency of CN compounds. Inhibition constants of CN compounds were in the range of 1.2–9.6 µM where the most potent was the compound CN 2Cl-6F followed by CN 2F and CN 4F (Table 1). Disubstituted *ortho*- and *para*-positioned fluorine atoms on CN derivatives or a combination of fluorine atoms with another halogen on position 6, have slightly better inhibition potency than corresponding CD derivatives. Generally, the affinity of BChE (1/*K*_i_) toward fluorinated CN derivatives is in the range of that determined for non-fluorinated CN’s [10].

All compounds inhibited AChE activity with *K*_i_ constants ranging from 3.9–69 µM (Table 1) with affinities about 1000 times lower than that of donepezil, a selective AChE inhibitor in use for the treatment of AD [28,29]. The most potent inhibitor was CN 4F with fluorine atom in *para*-position on the benzene ring. Inhibition potency of monosubstituted CD’s was generally 2.5–8 times lower compared to inhibition potency of disubstituted compounds and compounds with the trifluoromethoxy group. The affinity of fluorine derivatives of CN has changed very little, and no pattern can be deduced related to the determined differences in inhibition potency of CN’s and AChE. When inhibition potencies of compounds with *meta* (CN 3F) or *para* (CN 4F) positioned fluorine atom were compared to the inhibition potencies of CN’s with bromine or chlorine atoms in *para* positions, it can be concluded that substituents with the bulkier and less electronegative halogens are more favorable for binding into AChE active site [18].

#### 2.2.2. Inhibition by 10, 11-Dihydrocinchonine and 10, 11-Dihydrocinchonidine and Their Derivatives

The reduction of the 3-vinyl group to the 3-ethyl group affected the inhibition potency of compounds (Table 2).

For BChE, inhibition potencies of CD’s with the reduced 3-vinyl group were not changed or lowered compared to their non-reduced analogues (Table 1 and Table 2). In the case of DHCD, inhibition potency was similar to that of CD [18] while the inhibition potency of quaternary DHCD Bzl was 5 times lower than CD (Table 1 and Table 2). For CN’s with the 3-ethyl group, the effect was the opposite–reduction of the vinyl group increased the inhibition potency 4 or 3 times in the case of DHCN and DHCN Bzl, respectively, compared to their 3-vinyl analogues. The most potent CD-based inhibitors of BChE with the 3-ethyl group were: DHCD Bzl (a compound with no substituents on a benzene ring), DHCD 3F (a compound with the fluorine atom as *meta*-substituent), DHCD 3CF_3_ (a compound with *meta*-substituted trimethyl group). DHCD 3CF_3_ is a 5 times weaker inhibitor compared with DHCD and DHCD 3F. Introduction of other substituents on benzene ring decreased the inhibition compared to DHCD Bzl and DHCD 3F, which can be related to the increase of their lipophilicity. Inhibition potency of CN’s with the reduced 3-vinyl group was in the range of 0.9–2.2 µM, which indicates that introduction of the benzyl group with or without fluorine atoms and fluorine-containing groups did not contribute to the formation of new favorable binding interactions within the BChE active site.

All compounds with the reduced 3-vinyl group inhibited AChE with *K*_i_ constants in the 4.8–206 µM range. Interestingly, the lowest inhibition potency is determined for non-quaternary DHCD, while the highest potency was determined for quaternary non-substituted DHCD Bzl. Inhibition potencies of other compounds were in the 15–68 µM range. It seems that for binding of 3-ethyl derivatives in the AChE active site, compounds having CF_3_ or OCF_3_ substituents in the *ortho*-position on benzene ring are preferred. The inhibition potency of 3-ethyl derivatives was generally higher compared to their 3-vinyl analogues in AChE active site [18].

#### 2.2.3. Selectivity of Inhibition

The inhibition selectivity denoted as a selective index (SI) of compounds was calculated as the ratio of *K*_i_ constants determined for inhibition of AChE and BChE, Table 1 and Table 2. Generally, all synthesized compounds had a higher preference for BChE over AChE, and the highest selectivity was determined for CD 3F which displayed 533 times higher inhibition selectivity toward BChE over AChE. CD 3,5F followed with 123-times and DHCD 3F with 84-times higher inhibition selectivity towards BChE. As expected, the highest range of selectivity (SI = 0.77–533) was determined for CD’s while CN’s are generally less selective (SI = 2.1–33). In the case of 3-ethyl compounds, DHCN’s are generally more selective towards BChE than DHCD’s. The selectivity of here tested fluorinated CD’s and CN’s corresponds to that earlier determined for similar compounds [18] where the selectivity of *N*-*para*-bromobenzyl CD derivative was 510 times more selective to BChE than AChE.

#### 2.2.4. Stereoselectivity of Inhibition by *Pseudo-Enantiomers*

The ratio of *K*_i_ constants determined for CN’s and their corresponding *pseudo-enantiomers* CD’s (*K_i_*(CN’s/CD’s)) determined the stereoselectivity of inhibition for each cholinesterase, Table 3. When the ratio is > 1 it denotes that the enzyme has a higher affinity toward CD’s over CN’s. Generally, neither of the cholinesterases showed pronounced stereoselectivity toward one of the *pseudo-enantiomers*.

For fluorinated *pseudo-enantiomeric* pairs, BChE displayed 1.5–81 times higher affinity toward CD in ten enantiomeric pairs, Table 3, and the highest stereoselectivity was determined for *pseudo-enantiomeric* pair CN 3F /CD 3F where BChE had 81 times higher affinity toward CD *pseudo-enantiomer.* BChE had a 78 higher preference for *meta* disubstituted CD 3,5-F over CN 3,5-F. Those results pointed out the importance of *meta*-position of substituents in obtaining selectivity of BChE toward *pseudo-enantiomeric* pairs of tested compounds. It was observed that increasing the size of the *meta* substituent and changing its electronegativity, resulted in a selectivity decrease: 3F ≅ 3,5F > 3CF_3_ > 3OCF_3_. For the last pair (CN 3 OCF_3_/CD 3 OCF_3_) the reversal of stereoselectivity was determined.

AChE displayed 1.7–5 times higher affinity toward CD in 12 *pseudo-enantiomeric* pairs, where the highest stereoselectivity was determined for the trifluoromethoxy group in *meta*-position on the benzene ring (3OCF_3_). For *pseudo-enantiomeric* pairs with 3F and 4F substituents, AChE displayed 3- and 18-times higher affinity for CN *pseudo-enantiomers*. Selectivity of AChE toward *pseudo-enantiomeric* pairs of *meta*-positioned substituents changed from being three times more selective towards CN 3F *pseudo-enantiomer* and 5 times more selective towards CD *pseudo-enantiomers* in order: 3F > 3CF_3_ > 3OCH_3_. The *meta* substitution proved to be important for cholinesterases stereoselectivity. These results correspond to that determined earlier for *pseudo-enantiomers* of analogues with different substituents on benzene [18].

In the case of *pseudo-enantiomers* with the reduced 3-vinyl group, no pronounced preference for any of pseudo-enantiomers was observed, Table 4. BChE generally slightly preferred binding of CN derivatives of six (6/8) *pseudo-enantiomeric* pairs. 16 times higher affinity was found for unsubstituted DHCN over DHCD. On the contrary, AChE was up to 4.4 times more selective in the binding of DHCD *pseudo-enantiomers* (6/8). It is worth noting that both cholinesterases showed the same enantiomeric preference in the case of DHCN/ DHCD and DHCN Bzl/ DHCD Bzl, preferring DHCN derivatives. For all other compounds, the stereoselective preference of BChE and AChE were opposite.

### 2.3. In Silico Modelling

Since the final aim of the study was to explore the possibility of fluorinated CD’s and CN’s to be used as a drug for the treatment of diseases related to the changes in acetylcholine levels, particularly in CNS, we evaluated the drug-likeness of the compounds and their ability to cross the blood-brain barrier by active or passive transport.

Physico-chemical properties (molecular weight (MW), partition coefficient (logP), hydrogen bonds donors (HBD), hydrogen bond acceptors (HBA), number of rotatable bonds (RB) and polar surface area (PSA)) are presented in Table 5. They were equal for *pseudo-enantiomers* (CD and CN) and only results for CD derivatives are given. The physicochemical properties of 9 compounds were within the recommended range for orally active drugs in humans indicating their ability to pass the interface between blood and intestine by passive transport. The clogP, HBD, HBA and PSA values were in the desirable range in all tested compounds. In 14 compounds the MW value deviated from the recommended value, but it is relatively close to it. Thus, it can be assumed that those compounds would have a moderate possibility to passively transport through lipid membranes.

The ability of compounds to penetrate the blood–brain barrier, the log BBB value, was determined using BIOVIA ADMET Protocol (ADMET_BBB) implemented in Biovia Discovery Studio Client v18.1. According to the BIOVIA ADMET Protocol (ADMET_BBB) all of the tested compounds can penetrate the BBB by passive diffusion after oral administration, Table 6. Eight of the tested compounds are very high BBB penetrants having log BBB values within the range 0.773–0.894 (the concentration of the compound in the brain is at least five times higher than in the blood), while thirty-eight compounds are high BBB penetrants with logBBB within the range 0.256–0.671 (the ratio of compound concentration in the brain and blood is between 1:1 up to 5:1).

### 2.4. PCA and Activity/PES Model Established by Machine Learning

**Figure 3:**
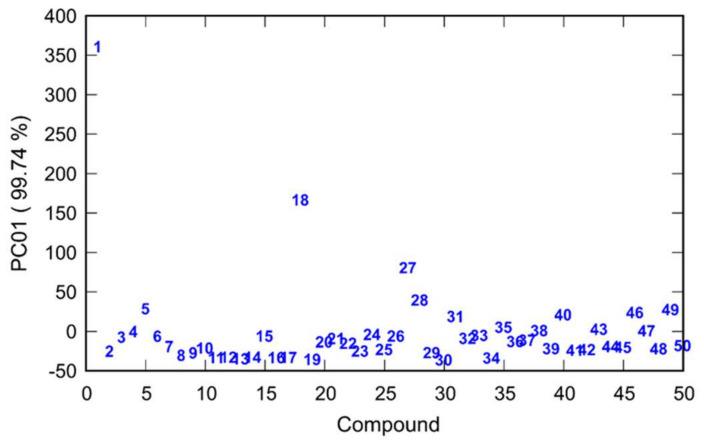
Classification of compounds 1–50 based on values calculated by PCA performed on the mean-centered covariance matrix of their inhibition of AChE and BChE. (Compound labels: 1 CD; 2 CD Bzl; 3 CD 2F; 4 CD 3F; 5 CD 4F; 6 CD 3CF_3_; 7 CD 4CF_3_; 8 CD 3,5F; 9 CD 3,4F; 10 CD 2,3F; 11 CD 2,4F; 12 CD 2,6F; 13 CD 3OCF_3_; 14 CD 4OCF_3_; 15 CD 2F-6CF_3_; 16 CD 4Br-2F; 17 CD 2Cl-6F; 18 DHCD; 19 DHCD Bzl; 20 DHCD 3F; 21 DHCD 4F; 22 DHCD 3CF_3_; 23 DHCD 4CF_3_; 24 DHCD 3OCF_3_; 25 DHCD 4OCF_3_; 26 CN; 27 CN Bzl; 28 CN 2F; 29 CN 3F; 30 CN 4F; 31 CN 3CF_3_; 32 CN 4CF_3_; 33 CN 3,5F; 34 CN 3,4F; 35 CN 2,3F; 36 CN 2,4F; 37 CN 2,6F; 38 CN 3OCF_3_; 39 CN 4OCF_3_; 40 CN 2F-6CF3; 41 CN 4Br-2F; 42 CN 2Cl-6F; 43 DHCN; 44 DHCN Bzl; 45 DHCN 3F; 46 DHCN 4F; 47 DHCN 3CF_3_; 48 DHCN 4CF_3_; 49 DHCN 3OCF_3_; 50 DHCN 4OCF_3_).

Principal component analysis was performed on the compounds’ inhibitory activities for BChE and AChE. The first principal component explained 99.74% of the total variance among the data assuring the proper description of the activities with only one-dimensional reduced space. All investigated compounds were classified according to their score values and the results are presented in Figure 3.

According to the results of PCA, the 1st principal component was the most important in describing the inhibition activity of the compounds. To establish a connection between inhibition of AChE and BChE and calculated potential energy surfaces of the compounds, an activity/PES model was created by using the first three principal components of the reduced PES data. The 1st principal component for inhibition of AChE and BChE was regressed on these three principal components.

An extensive machine learning procedure was utilized to build the best multivariate linear regression model. During the calculation all possible regression models were generated with determined B-matrices of coefficients (Equation (2)). These models were *cross-validated* using the *leave-one-out cross-validation* procedure [30]. The best possible regression models were determined based on following parameters: adjusted and predicted *R^2^* values, LOO-CV mean squared error and the number of variables in the models.

**Figure 4:**
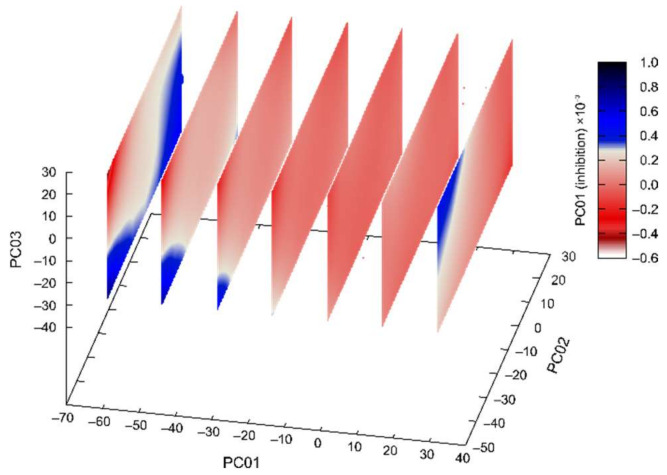
Machine learning determined the best multivariate regression model for fluorinated **CD**-derivatives inhibition of AChE and BChE in dependence on the first three principal components of compounds’ potential energy surfaces. (Spheres represent points in 3D reduced space, and the planes are cuts of the calculated polynomial regression model, for easier interpretation 4th-dimension is represented redundantly with the color and with the size of the spheres.).

For fluorinated **CD**-derivatives the best calculated 3D regression model is presented in Figure 4. The value of adjusted *R*^2^ was 0.9936 and predicted *R^2^* 0.9932 (*R^2^* was 0.9971). In this calculated model the high values of adjusted *R*^2^ and predicted *R^2^* ensures that there is no overfitting, and the models are thoroughly validated.

**Figure 5:**
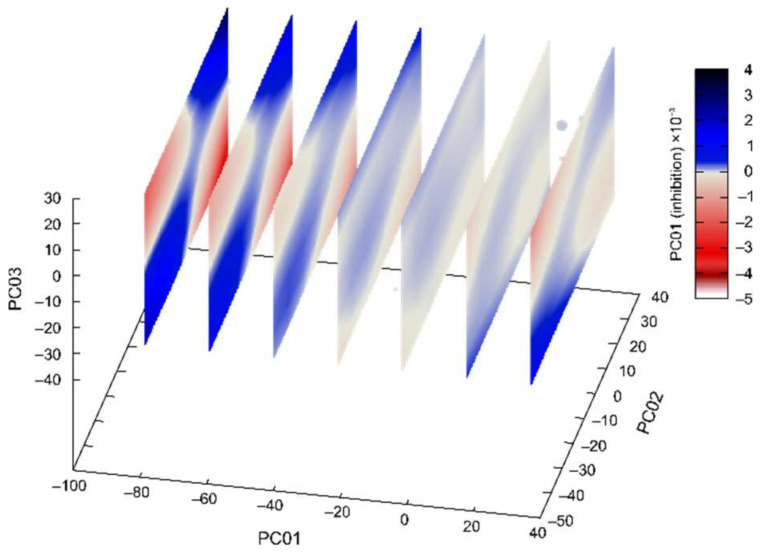
Machine learning determined the best multivariate regression model for fluorinated **CN**-derivatives inhibition of AChE and BChE in dependence on the first three principal components of compounds’ potential energy surfaces. (Spheres represent points in 3D reduced space, and the planes are cuts of the calculated polynomial regression model, for easier interpretation 4th-dimension is represented redundantly with the color and with the size of the spheres.).

The best calculated 3D regression model for fluorinated **CN**-derivatives is presented in Figure 5. Again, the high values of adjusted *R*^2^ (0.9849) and predicted *R^2^* (0.9879) (*R^2^* was 0.9899) assure that there was no overfitting in the model and that the model was thoroughly validated.

Established activity/PES regression models can be used for the prediction of AChE and BChE inhibition for new similar compounds based solely on the reduced space of compounds’ potential energy surfaces obtained from ab initio molecular dynamics simulation.

## 3. Materials and Methods

### 3.1. Chemicals

Reagents and solvents for the preparation of compounds were purchased from Sigma-Aldrich (St. Louis, MO, USA), Fluka. The reactions were monitored by thin-layer chromatography plates coated with silica gel (Sigma-Aldrich, St. Louis, MO, USA). TLC plates were visualized by UV irradiation (254 nM) or by iodine fumes. 1D and 2D ^1^H and ^13^C NMR spectra were recorded on a Bruker Avance III HD 400 MHz/54 mM Ascend spectrometer (Bruker Optics Inc, Billerica, MA, USA). Chemical shifts are given in ppm downfield from tetramethylsilane (TMS) as an internal standard and coupling constants (*J*) in Hz. Splitting patterns are designated as s (singlet), d (doublet), dd (doublet of doublets), ddd (doublet of doublet of doublets), t (triplet), q (quartet) or m (multiplet). Quinoline hydrogen and carbon atoms are marked with an apostrophe. Benzene hydrogen and carbon atoms are marked with a double apostrophe. Melting points were determined on a Melting Point B-540 apparatus (Büchi, Essen, Germany) and are uncorrected. Optical rotations were measured on an Optical Activity AA-10 automatic polarimeter (Optical Activity Limited, Ramsey, Cambridgeshire, UK) at 22 °C. HPLC analyses were performed on Agilent 1260 series instrument equipped with a quaternary pump, autosampler, column compartment and diode array detector (DAD). HRMS analyses were carried out on Q Exactive™ Plus Hybrid Quadrupole-Orbitrap™ Mass Spectrometer. HPLC conditions: Zorbax Extend-C18 column, 4.6 mM × 250 mM, 5 μM pore size; column temperature 25 °C; flow rate 1.0 mL/min; mobile phase A: H_2_O; mobile phase B: CH_3_CN; linear gradient H_2_O:CH_3_CN 10/90/90/10/10% B in time intervals 0/10/15/20/25; the volume of injection 10 µL; UV detection at 290 nM.

### 3.2. Synthesis

10,11-dihydrocinchonidine (DHCD) and 10,11-dihydrocinchonine (DHCN) were prepared by reduction of cinchonidine or cinchonine [31]. Quaternary compounds were prepared by reaction of the alkaloid (CD, CN, DHCD, DHCN) (1 mmol) and appropriate bromide (1.05 mmol) in refluxing 2-propanol. The end of reaction was detected with thin layer chromatography (CHCl_3_:MeOH = 9:1). After cooling to 22 °C, the precipitated product was isolated by filtration and recrystallized from methanol/diethyl ether. The data for novel compounds is present below, data for other prepared compounds were reported previously [21,22,23,24,25,26,27,28].

***N*-(2-fluoro-6-trifluoromethylbenzyl)cinchonidinium bromide, CD 2F-6CF_3_**. White solid. Yield: 44%; m.p. 243 °C; [𝛼] = −153 ° (c 0.1; MeOH); *t*_r_ 7.61 min; FTIR *ν̃*/cm^−1^: 3647 (O–H) 3151 (C–H_Ar_) 1589 (C=N) 1136 (C–O) 777 (C–F); ^1^H NMR (400 MHz, MeOD-d_3_) δ/ppm: 1.35–1.45 (1 H, m, H7a) 1.84–1.94 (1 H, m, H5b) 2.05–2.11 (1 H, m, H4) 2.18–2.32 (2 H, m, H5a, H7b) 2.75 (1 H, br. s., H3) 3.52–3.60 (1 H, m, H2a) 3.68–3.74 (1 H, m, H6b) 4.02–4.09 (1 H, m, H6a) 4.48 (1 H, t, *J* = 11.13 Hz, H8) 4.97–5.03 (2 H, m, CH2) 5.16 (1 H, dt, *J* = 17.30; 1.13 Hz, H11a) 5.30 (1 H, d, *J* = 12.96 Hz, H11b) 5.69 (1 H, ddd, *J* = 17.24; 10.51; 6.72 Hz, H10) 6.62–6.66 (1 H, m, H9) 7.61 (1 H, dd, *J* = 8.31; 1.96 Hz, H4’’’) 7.65 (1 H, dd, *J* = 9.66; 1.83 Hz, H3’’) 7.77–7.83 (2 H, m, H6’, H7’) 7.83–7.89 (1 H, m, H5’’) 7.96 (1 H, d, *J* = 4.65 Hz, H3’) 8.12 (1 H, dd, *J* = 8.44; 1.10 Hz, H8’) 8.31 (1 H, dd, *J* = 8.31; 0.98 Hz, H5’) 8.94 (1 H, d, *J* = 4.65 Hz, H2’); ^13^C NMR (101 MHz, MeOD-d_3_) δ/ppm: 22.69 (C7) 26.10 (C5) 27.89 (C4) 39.34 (C3) 53.12 (CH2) 58.14 (C6) 62.24 (C2) 66.55 (C9) 69.90 (C8) 115.89 (C4’’, *J*_C-F_ = 14.1 Hz) 117.74 (C11) 121.47 (C3’’, *J*_C-F_ = 26.2 Hz,) 124.26 (C7’) 126.24 (C9’) 127.43 (C1’’, *J*_C-F_ = 10.4 Hz) 129.35 (C6’) 130.02 (C5’’, *J*_C-F_ = 3.4 Hz,) 130.52 (C5’) 131.36 (C8’) 138.20 (C6’’, *J*_C-F_ = 3.4 Hz) 138.81 (C10) 147.57 (C10’) 148.90 (C4’) 151.20 (C2’) 163.593 (C2’’, *J*_C-F_ = 253.6 Hz,); HRMS (Electrospray ionisation (ESI) m/z calcd for C_27_H_27_F_4_N_2_O^+^ = 471.2054, found 471.2056 (mass error Δm = 0.42 ppm).

***N*-(2-fluoro-4-bromobenzyl)cinchonidinium bromide, CD 2F-4-Br**. White solid. Yield: 67%; m.p. 237 °C; [𝛼] = −186 ° (c 0.1; MeOH); *t*_r_ 7.79 min; FTIR *ν̃*/cm^−1^: 3382 (O–H) 3153 (C–H_Ar_) 1605 (C=N) 1130 (C–O) 781 (C–F); ^1^H NMR (400 MHz, MeOD-d_3_) δ/ppm: 1.35–1.45 (1 H, m, H7a) 1.84–1.94 (1 H, m, H5b) 2.05–2.11 (1 H, m, H4) 2.18–2.32 (2 H, m, H5a, H7b) 2.75 (1 H, br. s., H3) 3.52–3.60 (1 H, m, H2a) 3.68–3.74 (1 H, m, H6b) 4.02–4.09 (1 H, m, H6a) 4.48 (1 H, t, *J* = 11.13 Hz, H8) 4.97–5.03 (2 H, m, CH2) 5.16 (1 H, dt, *J* = 17.30; 1.13 Hz, H11a) 5.30 (1 H, d, *J* = 12.96 Hz, H11b) 5.69 (1 H, ddd, *J* = 17.24; 10.51; 6.72 Hz, H10) 6.62–6.66 (1 H, m, H9) 7.61 (1 H, dd, *J* = 8.31; 1.96 Hz, H4’’) 7.65 (1 H, dd, *J* = 9.66; 1.83 Hz, H3’’) 7.77–7.83 (2 H, m, H6’, H7’) 7.83–7.89 (1 H, m, H5’’) 7.96 (1 H, d, *J* = 4.65 Hz, H3’) 8.12 (1 H, dd, *J* = 8.44; 1.10 Hz, H8’) 8.31 (1 H, dd, *J* = 8.31; 0.98 Hz, H5’) 8.94 (1 H, d, *J* = 4.65 Hz, H2’); ^13^C NMR (101 MHz, MeOD-d_3_) δ/ppm: 22.69 (C7) 26.10 (C5) 27.89 (C4) 39.34 (C3) 53.12 (CH2) 58.14 (C6) 62.24 (C2) 66.55 (C9) 69.90 (C8) 115.89 (C4’’, *J*_C-F_ = 14.1 Hz) 117.74 (C11) 121.47 (C3’’, *J*_C-F_ = 26.2 Hz) 124.26 (C7’) 126.24 (C9’) 127.43 (C1’’, *J*_C-F_ = 10.4 Hz) 129.35 (C8’) 130.02 (C5’’, *J*_C-F_ = 3.4 Hz) 130.52 (C5’) 131.36 (C6’) 138.20 (C6’’, *J*_C-F_ = 3.4 Hz) 138.81 (C10) 147.57 (C10’) 148.90 (C4’) 151.20 (C2’) 163.60 (C2’’, *J*_C-F_ = 253.6 Hz); HRMS (Electrospray ionisation (ESI) m/z calcd for C_26_H_27_BrFN_2_O^+^ = 481.1285, found 481.1289 (mass error Δm = 0.83 ppm).

***N*-(2-chloro-6-fluorobenzyl)cinchonidinium bromide, CD 2Cl-6F**. White solid. Yield: 34%; m.p. 240–241 °C; [𝛼] = −220 ° (c 0.1; MeOH); *t*_r_ 7.33 min; FTIR *ν̃*/cm^−1^: 3431 (O–H) 3127 (C–H_Ar_) 1596 (C=N) 1114 (C–O) 774 (C–F); ^1^H NMR (400 MHz, MeOD-d_3_) *δ*/ppm: 1.39–1.48 (1 H, m, H7a) 1.91 (1 H, t, *J* = 9.29 Hz, H5b) 2.07 (1 H, d, *J* = 2.93 Hz, H4) 2.22–2.33 (2 H, m, H7b, H5a) 2.78 (1 H, br. s., H3) 3.32–3.39 (1 H, m, H2a) 3.59–3.75 (2 H, m, H2b, H6a) 4.14 (1 H, t, *J* = 9.05 Hz, H6b) 4.63–4.72 (1 H, m, H8) 4.99–5.06 (2 H, m, CH2) 5.11 (1 H, d, *J* = 17.12 Hz, H11a) 5.52 (1 H, d, *J* = 13.20 Hz, H11b) 5.72 (1 H, ddd, *J* = 17.24; 10.39; 7.09 Hz, H10) 6.72 (1 H, s, H9) 7.39–7.45 (1 H, m, H4’’) 7.58 (1 H, d, *J* = 8.07 Hz, H3’’) 7.68 (1 H, td, *J* = 8.31; 6.11 Hz, H5’’) 7.81 (1 H, td, *J* = 7.64; 1.34 Hz, H7’) 7.87 (1 H, td, *J* = 7.64; 1.34 Hz, H6’) 7.98 (1 H, d, *J* = 4.65 Hz, H3’) 8.11–8.15 (1 H, m, H8’) 8.31 (1 H, d, *J* = 8.31 Hz, H5’) 8.96 (1 H, d, *J* = 4.65 Hz, H2’); ^13^C NMR (101 MHz, MeOD-d_3_) *δ*/ppm: 22.79 (C7) 26.29 (C5) 27.55 (C4) 39.67 (C3) 53.44 (CH2, *J*_C-F_ = 3.4 Hz) 57.09 (C6) 62.93 (C2, *J*_C-F_ = 4.7 Hz) 66.71 (C9) 70.45 (C8) 116.28 (C1’’, *J*_C-F_ = 16.7 Hz) 116.90 (C5’’, *J*_C-F_ = 23.7 Hz) 117.73 (C11) 121.54 (C7’) 124.12 (C3’) 126.32 (C9) 128.45 (C3’’, *J*_C-F_ = 3.3 Hz) 129.27 (C8’) 130.55 (C5’) 131.39 (C6’) 135.38 (C4’’, *J*_C-F_ = 10.7 Hz) 138.87 (C10) 139.56 (C2’’) 147.67 (C10’) 148.92 (C4’) 151.22 (C2’) 164.50 (C6’’, *J*_C-F_ = 252.5 Hz); HRMS (Electrospray ionisation (ESI) m/z calcd for C_26_H_27_ClFN_2_O^+^ = 437.1790, found 437.1791 (mass error Δm = 0.23 ppm).

***N*-(2,3-difluorobenzyl)cinchoninium bromide, CN 2,3F**. White solid. Yield: 70%; m.p. 239 °C; [𝛼] = +168 ° (c 0.1; MeOH); *t*_r_ 7.32 min; FTIR *ν̃*/cm^−1^: 3442 (O–H) 3113 (C–H_Ar_) 1587 (C=N) 1132 (C–O) 777 (C–F); ^1^H NMR (400 MHz, MeOD-d_3_) *δ*/ppm: 1.04–1.14 (1 H, m, H7a) 1.85–1.97 (3 H, m, H4, H5) 2.44–2.54 (1 H, m, H7b) 2.68 (1 H, q, *J* = 8.72 Hz, H3) 3.24–3.27 (1H, m, H2a) 3.46–3.55 (1 H, m, H6a) 3.92–4.03 (1 H, m, H2b) 4.11 (1 H, t, *J* = 9.54 Hz, H8) 4.46 (1 H, ddd, *J* = 11.98; 8.80; 2.69 Hz, H6b) 5.08–5.19 (1 H, m, CH2a) 5.21–5.31 (3 H, m, CH2b; H11) 5.99–6.14 (1 H, m, H10) 6.62–6.67 (1 H, m, H9) 7.36–7.47 (1 H, m, H6’’) 7.52–7.62 (1 H, m, H5’’) 7.63–7.68 (1 H, m, H4’’) 7.80–7.90 (2 H, m, H3’; H7’) 7.95–8.01 (1 H, m, H6’) 8.12 (1 H, dd, *J* = 8.31; 1.22 Hz, H8’) 8.32–8.40 (1 H, m, H5’) 8.96 (1 H, d, *J* = 4.65 Hz, H2’); ^13^C NMR (101 MHz, MeOD-d_3_) *δ*/ppm: 20.92 (C7) 23.31 (C5) 26.78 (C4) 37.56 (C3) 55.18 (C6) 56.03 (C2) 56.78 (CH2) 65.61 (C9) 67.85 (C8) 116.61 (C11) 117.37 (C1’’, *J*_C-F_ = 10.24 Hz) 119.83 (C3’) 119.97 (C4’’, *J*_C-F_ = 16.81 Hz) 122.94 (C7’) 124.75 (C9’) 125.24 (C5’’, *J*_C-F_ = 7.42 Hz) 127.77 (C6’) 128.88 (C5’) 129.83 (C8’) 130.25 (C6’’, *J*_C-F_ = 3.4 Hz) 136.15 (C10) 145.76 (C10’) 147.35 (C4’) 149.65 (C2’); HRMS (Electrospray ionisation (ESI) m/z calcd for C_26_H_27_F_2_N_2_O^+^ = 421.2086, found 421.2086 (mass error Δm = 0.00 ppm).

***N*-(2,4-difluorobenzyl)cinchoninium bromide, CN 2,4F**. White solid. Yield: 51%; m.p. 230–231 °C; [𝛼] = +155 ° (c 0.1; MeOH); *t*_r_ 7.31 min; FTIR *ν̃*/cm^−1^: 3438 (O–H) 3104 (C–H_Ar_) 1580 (C=N) 1131 (C–O) 777 (C–F); ^1^H NMR (400 MHz, MeOD-d_3_) *δ*/ppm: 1.02–1.13 (1 H, m, H7a) 1.83–1.99 (3 H, m, H5; H4 ) 2.42–2.51 (1 H, m, H7b) 2.67 (1 H, q, *J* = 8.56 Hz, H3) 3.42–3.52 (1 H, m, H2a) 3.93–4.01 (1 H, m, H6b) 4.09 (1 H, t, *J* = 9.54 Hz, H8) 4.40–4.49 (1 H, m, H6a) 5.09 (1 H, d, *J* = 13.20 Hz, CH2a) 5.19 (1 H, d, *J* = 12.72 Hz, CH2b) 5.25–5.31 (2 H, m, H11) 6.06 (1 H, ddd, *J* = 17.42; 10.21; 7.34 Hz, H10) 6.64 (1 H, d, *J* = 2.20 Hz, H9) 7.19–7.29 (2 H, m, ArH’’) 7.79–7.97 (4 H, m, H6’; H7’, ArH’’) 8.12 (1 H, dd, *J* = 8.31; 1.22 Hz, H8’) 8.35–8.40 (1 H, m, H5’) 8.95 (1 H, d, *J* = 4.40 Hz, H2’); ^13^C NMR (101 MHz, MeOD-d_3_) *δ*/ppm: 20.89 (C7) 23.30 (C5) 26.88 (C4) 37.56 (C3) 54.97 (CH2) 55.94 (C6) 56.54 (C2) 65.59 (C9) 67.65 (C8) 104.56 (C3’’, *J*_C-F_ = 25.3 Hz) 111.34 (C1’’, *J*_C-F_ = 14.9; 3.9 Hz) 112.39 (C5’’, *J*_C-F_ = 22.7; 3.8 Hz) 116.57 (C11) 119.82 (C3’) 123.01 (C7’) 124.75 (C9’) 127.79 (C6’) 128.84 (C5’) 129.81 (C8’) 136.22 (C10’) 136.87 (C6’’, *J*_C-F_ = 10.0; 3.4 Hz) 145.83 (C4’) 147.33 (C10’) 149.63 (C2’) 162.53 (C4’’, *J*_C-F_ = 205.5; 12.6 Hz) 166.21 (C2’’, *J*_C-F_ = 219.2; 12.6 Hz); HRMS (Electrospray ionisation (ESI) m/z calcd for C_26_H_27_F_2_N_2_O^+^ = 421.2086, found 421.2086 (mass error Δm = 0.00 ppm).

***N*-(2,6-difluorobenzyl)cinchoninium bromide, CN 2,6F**. White solid. Yield: 36%; m.p. 242 °C; [𝛼] = +54 ° (c 0.1; MeOH); *t*_r_ 7.40 min; FTIR *ν̃*/cm^−1^: 3446 (O–H) 3102 (C–H_Ar_) 1579 (C=N) 1134 (C–O) 776 (C–F); ^1^H NMR (400 MHz, MeOD-d_3_) *δ*/ppm: 1.08–1.17 (1 H, m, H7a) 1.89–2.07 (m, 3 H, H5; H4) 2.50 (1 H, t, *J* = 11.74 Hz, H7b) 2.71 (1 H, q, *J* = 8.56 Hz, H3) 3.35–3.56 (2 H, m, H2a; H6b) 3.90–4.01 (1 H, m, H2b) 4.17 (1 H, t, *J* = 9.29 Hz, H8) 4.50 (1 H, t, *J* = 9.17 Hz, H6a) 4.98–5.07 (1 H, m, CH2a) 5.22–5.38 (3 H m, CH2b; H11) 6.10 (1 H, ddd, *J* = 17.24, 10.39, 7.34 Hz, H10) 6.69 (1 H, br. s., H9) 7.32 (2 H, t, *J* = 8.80 Hz, H6’; H7’) 7.72–7.81 (1 H, m, ArH’’) 7.81–7.93 (2 H, m, ArH’’) 7.99 (1 H, d, *J* = 4.40 Hz, H3’) 8.16 (1 H, d, *J* = 8.31 Hz, H8’) 8.34 (1 H, d, *J* = 8.07 Hz, H5’) 8.99 (1 H, d, *J* = 4.65 Hz, H2’); ^13^C NMR (101 MHz, MeOD-d_3_) *δ*/ppm: 20.96 (C7) 23.39 (C5) 26.52 (C4) 37.66 (C3) 51.40 (C6) 55.13 (C2) 56.67 (CH2, *J*_C-F_ = 3.26 Hz) 65.69 (C9) 67.80 (C8) 112.17 (C3’’) 112.39 (C6’’) 116.53 (C1’’) 116.60 (C11) 119.86 (C3’) 122.80 (C7’) 124.79 (C9’) 127.67 (C6’) 128.89 (C5’) 129.85 (C8’) 134.25 (C4’’, *J*_C-F_ = 11 Hz) 136.22 (C10) 145.73 (C1’) 147.37 (C4’) 149.66 (C2’) 161.06 (C2’’) 163.56 (C6’’); HRMS (Electrospray ionization (ESI) m/z calcd for C_26_H_27_F_2_N_2_O^+^ = 421.2086, found 421.2087 (mass error Δm = 0.24 ppm).

***N*-(3-trifluoromethoxybenzyl)cinchoninium bromide, CN 3OCF_3_**. White solid. Yield: 86%; m.p. 218 °C; [𝛼] = +103 ° (c 0.1; MeOH); *t*_r_ 8.23 min; FTIR *ν̃*/cm^−1^: 3443 (O–H) 3010 (C–H_Ar_) 1582 (C=N) 1130 (C–O) 779 (C–F); ^1^H NMR (400 MHz, MeOD-d_3_) *δ*/ppm: 1.08 (1 H, ddd, *J* = 13.14; 8.86; 4.89 Hz, H7a) 1.79–1.96 (3 H, m, H4; H5) 2.43–2.53 (1 H, m, H7b) 2.66 (1 H, q, *J* = 8.56 Hz, H3) 3.11 (1 H, dt, *J* = 11.62; 9.35 Hz, H2b) 3.57–3.66 (1 H, m, H6a) 3.93–4.09 (2 H, m, H2a; H8) 4.46 (1 H, ddd, *J* = 12.10; 8.80; 2.81 Hz, H6b) 5.07 (1 H, d, *J* = 12.47 Hz, CH2a) 5.21 (1 H, d, *J* = 12.47 Hz, CH2b) 5.25–5.32 (2 H, m, H11) 6.06 (1 H, ddd, *J* = 17.24; 10.39; 7.09 Hz, H10) 6.62 (1 H, d, *J* = 2.20 Hz, H9) 7.51–7.56 (1 H, m, H3’) 7.70 (1 H, t, *J* = 8.07 Hz, H7’) 7.76–7.88 (4 H, m, ArH’’) 7.95–7.98 (1 H, m, H6’) 8.10–8.14 (1 H, m, H8’) 8.36 (1 H, dd, *J* = 8.31; 1.22 Hz, H5’) 8.95 (1 H, d, *J* = 4.65 Hz, H2’); ^13^C NMR (101 MHz, MeOD-d_3_) *δ*/ppm: 22.45 (C7) 24.82 (C5) 28.58 (C4) 38.99 (C3) 56.30 (C6) 58.26 (C2) 63.73 (CH2) 67.07 (C9) 69.50 (C8) 118.14 (C11) 123.33 (C3’) 124.37 (C8’) 126.28 (C9’) 129.34 (C6’) 130.40 (C5’) 131.32 (C7’) 131.34 (C1’’) 131.39 (C4’’) 132.32 (C6’’) 134.00 (C2’’ C5’’) 137.72 (C10) 147.37 (C10’) 148.89 (C4’) 150.97 (C3’’) 151.18 (C2’); HRMS (Electrospray ionization (ESI) m/z calcd for C_26_H_27_F_3_N_2_O_2_^+^ = 469.2097, found 469.2101 (mass error Δm = 0.85 ppm).

***N*-(2-fluoro-6-trifluoromethylbenzyl)cinchoninium bromide, CN 2F-6CF_3_**. White solid. Yield: 24%; m.p. 237 °C; [𝛼] = +114 ° (c 0.1; MeOH); *t*_r_ 8.53 min; FTIR *ν̃*/cm^−1^: 3420 (O–H) 3112 (C–H_Ar_) 1577 (C=N) 1127 (C–O) 774 (C–F); ^1^H NMR (400 MHz, MeOD-d_3_) *δ*/ppm: 1.05–1.12 (1 H, m, H7a) 1.85–2.01 (3 H, m, H4; H5) 2.43–2.55 (1 H, m, H7b) 2.69 (1 H, q, *J* = 8.72 Hz, H3) 3.88–3.99 (1 H, m, H2b) 4.15 (1 H, t, *J* = 9.66 Hz, H6b) 4.40–4.51 (1 H, m, H6a) 5.02 (1 H, d, *J* = 12.96 Hz, CH2a) 5.20–5.34 (3 H, m, H11, CH2b) 6.01–6.14 (1 H, m, H10) 6.67 (1 H, d, *J* = 2.45 Hz, H9) 7.29 (2 H, t, *J* = 8.56 Hz, H6’; H7’) 7.68–7.78 (2 H, m,) 7.79–7.90 (2 H, m,) 7.97 (1 H, d, *J* = 4.65 Hz, H3’) 8.13 (1 H, dd, *J* = 8.44; 1.10 Hz, H8’) 8.33 (1 H, d, *J* = 8.56 Hz, H5’) 8.91–9.00 (1 H, m, H2’); ^13^C NMR (101 MHz, MeOD-d_3_) *δ*/ppm: 21.17 (C7) 23.39 (C5) 26.52 (C4) 37.66 (C3) 53.56 (C6) 55.13 (C2) 57.12 (CH_2_, *J*_C-F_ = 8.8 Hz) 65.69 (C9) 68.44 (C8) 116.52 (C11) 119.86 (C3′ C7′) 120.99 (C5′’, *J*_C-F_ = 25.6 Hz) 122.80 (C4′’) 124.79 (C9′) 127.67 (C6′) 128.89 (C5′) 129.88 (C8′) 134.06 (C3′’, *J*_C-F_ = 10.8 Hz) 136.22 (C10) 145.73 (C10′) 147.37 (C4′) 149.66 (C2′) 162.92 (C2’’, *J*_C-F_ = 255 Hz); HRMS (Electrospray ionization (ESI) m/z calcd for C_27_H_27_F_4_N_2_O^+^ = 471.2054, found 471.2054 (mass error Δm = 0.00 ppm).

***N*-(2-fluoro-4-bromobenzyl)cinchoninium bromide, CN 2F-4Br**. White solid. Yield: 40%; m.p. 234-235 °C; [𝛼] = +94 ° (c 0.1; MeOH); *t*_r_ 7.83 min; FTIR *ν̃*/cm^−1^: 3416 (O–H) 3110 (C–H_Ar_) 1587 (C=N) 1131 (C–O) 776 (C–F); ^1^H NMR (400 MHz, MeOD-d_3_) *δ*/ppm: 1.03–1.15 (1 H, m, H7a) 1.86–1.96 (3 H, m, H4; H5) 2.41–2.51 (1 H, m, H7b) 2.67 (1 H, q, *J* = 8.56 Hz, H3) 3.22-3.27 (1H, m, H2a) 3.41–3.50 (1 H, m, H6b) 3.91–3.99 (1 H, m, H2b) 4.09 (1 H, t, *J* = 9.54 Hz, H8) 4.39–4.48 (1 H, m, H6a) 5.07 (1 H, d, *J* = 12.96 Hz, CH2a) 5.18 (1 H, d, *J* = 12.72 Hz, CH2b) 5.24–5.30 (2 H, m, H11) 6.00–6.11 (1 H, m, H10) 6.63 (1 H, d, *J* = 2.20 Hz, H9) 7.61 (1 H, dd, *J* = 8.31; 1.71 Hz, H7’) 7.66 (1 H, dd, *J* = 9.78; 1.96 Hz, H6’) 7.76–7.84 (2 H, m, H3’’; H6’’) 7.84–7.89 (1 H, m, H5’’) 7.96 (1 H, d, *J* = 4.65 Hz, H3’) 8.10–8.14 (1 H, m, H8’) 8.36 (1 H, d, *J* = 7.83 Hz, H5’) 8.95 (1 H, d, *J* = 4.65 Hz, H2’); ^13^C NMR (101 MHz, MeOD-d_3_) *δ*/ppm: 22.43 (C5) 24.84 (C7) 28.37 (C4) 39.09 (C3) 56.65 (C6, *J* = 3.54 Hz) 57.53 (C2) 58.17 (CH_2_, *J*_C-F_ = 1.48 Hz) 67.13 (C9) 69.32 (C8) 115.86 (C4’’, *J*_C-F_ = 14.23 Hz) 118.13 (C11) 121.34 (C3’’, *J*_C-F_ = 1.81 Hz) 121.60 (C3’) 124.47 (C7’) 126.29 (C9’) 127.34 (C1’’, *J*_C-F_ = 10.5 Hz) 129.31 (C6’) 130.01 (C5’’, *J*_C-F_ = 3.5 Hz) 130.42 (C5’) 131.36 (C8’) 137.73 (C10) 138.10 (C6’’, *J*_C-F_ = 2.7 Hz) 147.31 (C4’) 148.89 (C10’) 151.19 (C2’) 162.29 (C2’’, *J*_C-F_ = 254.08 Hz); HRMS (Electrospray ionization (ESI) m/z calcd for C_26_H_27_FBrN_2_O^+^ = 481.1285, found 481.1286 (mass error Δm = 0.21 ppm).

***N*-(2-chloro-6-fluorobenzyl)cinchoninium bromide, CN 2Cl-6F**. White solid. Yield: 65%; m.p. 241 °C; [𝛼] = +171 ° (c 0.1; MeOH); *t*_r_ 7.30 min; FTIR *ν̃*/cm^−1^: 3419 (O–H) 3115 (C–H_Ar_) 1567 (C=N) 1124 (C–O) 775 (C–F); **^1^H NMR** (400 MHz, MeOD-*d*3) *δ*/ppm: 1.08 (1 H, t, *J* = 12.96 Hz, H5b) 1.83–2.00 (3 H, m, H4; H7) 2.41–2.54 (1 H, m, H5a) 2.68 (1 H, q, *J* = 8.64 Hz, H3) 3.45–3.53 (2 H, m, H2) 4.05 (1 H, br. s., H6b) 4.18 (1 H, t, *J* = 9.54 Hz, H8) 4.54–4.63 (1 H, m, H6a) 5.11 (1 H, d, *J* = 12.96 Hz, CH2a) 5.24–5.30 (2 H, m, CH2b; H11a) 5.44 (1 H, d, *J* = 13.94 Hz, H11b) 6.07 (1 H, ddd, *J* = 17.18; 10.45; 7.34 Hz, H10) 6.68–6.72 (1 H, m, H9) 7.41 (1 H, t, *J* = 8.80 Hz, H6’) 7.58 (1 H, d, *J* = 8.07 Hz, H7’) 7.79–7.90 (2 H, m, ArH’’) 7.96–8.00 (1 H, m, ArH’’) 8.13 (1 H, d, *J* = 7.83 Hz, H8’) 8.33 (1 H, d, *J* = 8.07 Hz, H5’) 8.96 (1 H, d, *J* = 4.65 Hz, H2’); **^13^C NMR** (101 MHz, MeOD-*d*3) *δ*/ppm: 22.60 (C7) 25.05 (C5) 27.92 (C4) 39.30 (C3) 56.34 (CH2) 57.00 (C6) 58.46 (C2) 67.35 (C9) 69.52 (C8) 116.32 (C1’’, *J*_C-F_ = 16.9 Hz) 116.91 (C5’’, *J*_C-F_ = 23.5 Hz) 118.10 (C11) 121.39 (C3’) 124.33 (C7’) 126.35 (C9’) 128.47 (C5’’, *J*_C-F_ = 3.3 Hz) 129.22 (C6’) 130.44 (C5’) 131.39 (C8’) 135.34 (C4’’, *J*_C-F_ = 10.5 Hz) 137.83 (C10) 139.58 (C2’’, *J*_C-F_ = 4.1 Hz) 147.39 (C10’) 148.91 (C4’) 151.21 (C2’) 164.52 (C6’’, *J*_C-F_ = 239.9 Hz); HRMS (Electrospray ionization (ESI) m/z calcd for C_26_H_27_ClFN_2_O^+^ = 437.1790, found 437.1791 (mass error Δm = 0.23 ppm).

***N*-(4-fluorobenzyl)-10,11-dihydrocinchonidinium bromide, DHCD 4F**. White solid. Yield: 87%; m.p. 220.6 °C; [𝛼] = –180 ° (c 0.1; MeOH); *t*_r_ 7.45 min; FTIR *ν̃*/cm^−1^: 3435 (O–H) 3144 (C–H) 1167 (C–N) 753 (C–F); ^1^H NMR (400 MHz, MeOD-d_3_) *δ*/ppm: 0.82 (3H, t, *J* = 7.4 Hz, H11) 1.21–1.45 (3H, m, H5b, H10) 1.79–1.89 (2H, m, H4, H7a) 2.07 (1H, d, *J* = 2.5 Hz, H7) 2.24–2.32 (2H, m, H2a, H3) 3.36–3.45 (2H, m, H2b, H6a) 4.00 (1H, t, *J* = 9.2 Hz, H6) 4.42–4.48 (1H, m, H8) 4.98 (1H, d, *J* = 12.5 Hz, CH2a) 5.20 (1H, d, *J* = 12.5 Hz, CH2b) 6.67 (s, 1H, H9) 7.31–7.35 (2H, t, *J* = 8.7 Hz, H3’, H7’) 7.77–7.90 (4H, m, H2˝, H3˝, H5˝, H6˝) 8.00 (1H, d, *J* = 4.5 Hz, H6’) 8.15 (1H, dd, *J* = 8.5; 1.2 Hz, H5’) 8.32 (1H, d, *J* = 8.0 Hz, H8’) 8.98 (1H, d, *J* = 4.6 Hz, H2’); ^13^C NMR (101 MHz, MeOD-d3) *δ*/ppm: 10.28 (C11) 20.73 (C10) 24.20 (C4) 25.05 (C5) 26.07 (C7) 35.95 (C3) 51.28 (C2) 62.54 (C6) 62.89 (CH_2_) 64.87 (C8) 68.29 (C9) 115.77 (C3’) 115.99 (C6’) 120.02 (C5˝) 122.71 (C7’) 123.57 (C9’) 127.80 (C8’) 128.99 (C6˝) 129.78 (C5’) 135.71 (C2˝) 135.80 (C3˝) 146.22 (C10’) 147.37 (C4’) 149.66 (C2’) 162.86 (C1˝) 165.35 (C4˝); HRMS (Electrospray ionization (ESI) m/z calcd for C_26_H_30_FN_2_O^+^ = 405.2337, found 405.2337 (mass error Δm = 0.00 ppm).

***N*-(3-trifluoromethylbenzyl)-10,11-dihydrocinchonidinium bromide, DHCD 3CF_3_**. White solid. Yield: 58%; m.p. 213.8 °C; [𝛼] = –171 ° (c 0.1; MeOH); *t*_r_ 8.16 min; FTIR *ν̃*/cm^−1^: 3432 (O–H) 3171 (C–H) 1131 (C–N) 776 (C–F); ^1^H NMR (400 MHz, MeOD-d_3_) *δ*/ppm: 0.81 (3H, t, *J* = 7.4 Hz, H11) 1.20–1.38 (m, 2H, H5a, H7b) 1.41–1.47 (1H, m, H4) 1.81–1.91 (2H, m, H10) 2.08 (1H, d, *J* = 2.6 Hz, H7) 2.23–2.34 (2H, m, H3, H5a) 3.44–3.46 (2H, m, H2) 4.04 (1H, t, *J* = 9.2 Hz, H6b) 4.51–4.55 (1H, m, H8) 5.10 (1H, d, *J* = 12.5 Hz, CH2a) 5.32 (1H, d, *J* = 12.5 Hz, CH2b) 6.68 (s, 1H, H9) 7.80–7.94 (4H, m, H2˝, H4˝, H5˝, H6˝) 8.00 (1H, d, *J* = 4.6 Hz, H6’) 8.05–8.15 (2H, m, H3’, H7’) 8.15 (1H, dd, *J* = 8.5; 1.4, Hz, H5’) 8.33 (1H, d, *J* = 4.6 Hz, H8’) 8.98 (1H, d, *J* = 4.6 Hz, H2’); ^13^C NMR (101 MHz, MeOD-d3) *δ*/ppm: 10.27 (C11) 20.76 (C10) 24.11 (C4) 25.03 (C5) 26.00 (C7) 35.91 (C3) 51.59 (C2) 62.70 (C6) 62.90 (CH2) 64.89 (C8) 68.59 (C9) 120.02 (C3’) 122.80 (C6’) 124.76 (C9’) 127.06 (C5˝) 127.84 (C7’) 128.85 (C8˝) 128.98 (C8’) 129.80 (C6˝) 129.95 (C5’) 130.10 (C2˝) 131.03 (C1˝) 131.36 (C3˝) 137.35 (C4˝) 146.13 (C10’) 147.38 (C4’) 149.66 (C2’); HRMS (Electrospray ionisation (ESI) m/z calcd for C_27_H_30_F_3_N_2_O^+^ = 455.2305, found 455.2305 (mass error Δm = 0.00 ppm).

***N*-(3-trifluoromethoxybenzyl)-10,11-dihydrocinchonidinium bromide, DHCD 3OCF_3_**. White solid. Yield: 50%; m.p. 212.7 °C; [𝛼] = –150 ° (c 0.1; MeOH); *t*_r_ 8.16 min; FTIR *ν̃*/cm^−1^: 3444 (O–H) 3182 (C–H) 1155 (C–N) 779 (C–F); ^1^H NMR (400 MHz, MeOD-d3) *δ*/ppm: 0.81 (3H, t, *J* = 7.4 Hz, H11) 1.18–1.37 (2H, m, H5a, Hb7) 1.41–1.47 (1H, m, H4) 1.82–1.92 (2H, m, H10) 2.08 (1H, s, H7) 2.23–2.34 (2H, m, H3, H5b) 3.37–3.53 (3H, m, H2, H6b) 4.03 (1H, t, *J* = 9.0 Hz, H6) 4.48–4.52 (1H, m, H8) 5.04 (1H, d, *J* = 12.4 Hz, CH2a) 5.25 (1H, d, *J* = 12.4 Hz, CH2a) 6.66 (1H, s, H9) 7.55 (1H, d, *J* = 8.1 Hz, H3’) 7.70–7.90 (5H, m, H2˝, H4˝, H5˝, H6˝, H7’) 7.99 (1H, d, *J* = 4.5 Hz, H6’) 8.1 (1H, d, *J* = 4.2 Hz, H5’) 8.32 (1H, d, *J* = 8.0 Hz, H8’) 8.98 (1H, d, *J* = 4.6 Hz, H2’); ^13^C NMR (101 MHz, MeOD-d3) *δ*/ppm: 10.25 (C11) 20.77 (C10) 24.17 (C4) 25.04 (C5) 26.04 (C7) 35.92 (C3) 51.61 (C2) 62.73 (C6) 62.85 (CH2) 64.88 (C8) 68.58 (C9) 119.23 (C1˝) 120.02 (C3’) 122.77 (C6’) 122.82 (C4˝) 124.76 (C9’) 126.08 (C5˝) 127.82 (C7’) 128.98 (C8’) 129.79 (C6˝) 129.88 (C8˝) 130.78 (C5’) 132.39 (C2˝) 146.13 (C10’) 147.38 (C4’) 149.38 (C3˝) 149.66 (C2’); HRMS (Electrospray ionisation (ESI) m/z calcd for C_27_H_30_F_3_N_2_O_2_^+^ = 471.2254, found 471.2257 (mass error Δm = 0.64 ppm).

***N*-(4-trifluoromethoxybenzyl)-10,11-dihydrocinchonidinium bromide, DHCD 4OCF_3_**. White solid. Yield: 39%; m.p. 228.3 °C; [𝛼] = –155 ° (c 0.1; MeOH); *t*_r_ 8.43 min; FTIR *ν̃*/cm^−1^: 3432 (O–H) 3144 (C–H) 1161 (C–N) 788 (C–F); ^1^H NMR (400 MHz, MeOD-d3) *δ*/ppm: 0.82 (3H, t, *J* = 7.4 Hz, H11) 1.18–1.39 (m, 2H, H5a, H7b) 1.42–1.45 (1H, m, H4) 1.81–1.92 (2H, m, H10) 2.08 (1H, s, H7a) 2.22–2.33 (2H, m, H3, H5b) 3.36–3.49 (3H, m, H2, H2a, H6b) 4.03 (1H, t, *J* = 9.1 Hz, H6a) 4.46–4.51 (1H, m, H8) 5.03 (1H, d, *J* = 12.5 Hz, CH2a) 5.23 (1H, d, *J* = 12.5 Hz, CH2b) 6.67 (1H, s, H9) 7.51 (2H, d, *J* = 8.1 Hz, H3’, H7’) 7.81–7.90 (4H, m, H2˝, H3˝, H5˝, H6˝) 8.00 (1H, d, *J* = 4.6 Hz, H6’) 8.15 (1H, d, *J* = 4.2 Hz, H8’) 8.33 (1H, d, *J* = 8.0 Hz, H5’) 8.98 (1H, d, *J* = 4.6 Hz, H2’); ^13^C NMR (101 MHz, MeOD-d3) *δ*/ppm: 10.29 (C11) 20.77 (C10) 24.16 (C4) 25.04 (C5) 26.06 (C7) 35.93 (C3) 51.44 (C2) 62.66 (C6; CH_2_) 64.91 (C8) 68.41 (C9) 119.23 (C1˝) 120.04 (C3’) 121.17 (C3˝; C5˝) 122.77 (C6’) 124.75 (C9’) 126.55 (C8˝) 127.83 (C7’) 128.98 (C8’) 129.79 (C5’) 135.57 (C2˝; C6˝) 146.18 (C10’) 147.37 (C4’) 149.66 (C2’) 150.68 (C4˝); HRMS (Electrospray ionisation (ESI) m/z calcd for C_27_H_30_F_3_N_2_O_2_^+^ = 471.2254, found 471.2257 (mass error Δm = 0.64 ppm).

***N*-(3-fluorobenzyl)-10,11-dihydrocinchoninium bromide, DHCN 3F**. White solid. Yield: 48%; m.p. 223.7 °C; [𝛼] = +155 ° (c 0.1; MeOH); *t*_r_ 7.45 min; FTIR *ν̃*/cm^−1^: 3400 (O–H) 3162 (C–H) 1152 (C–N) 782 (C–F); ^1^H NMR (400 MHz, MeOD-d3) *δ*/ppm: 0.96 (3H, t, *J* = 7.4 Hz, H11) 1.04–1.11 (1H, m, H5a) 1.58–1.71 (2H, m, H10) 1.79–1.91 (3H, m, H4, H5b, H7a) 1.97 (1H, s, H7b) 2.46 (1H, t, *J* = 11.9 Hz, H3) 3.07–3.14 (1H, m, H2a) 3.64 (1H, t, *J* = 11.1 Hz, H6b) 3.93–4.06 (2H, m, H6a, H2b) 4.15–4.21 (1H, m, H8) 5.02 (1H, d, *J* = 12.4 Hz, CH2a) 5.15 (1H, d, *J* = 12.4 Hz, CH2b) 6.61 (1H, s, H9) 7.34–7.40 (1H, m, H6˝) 7.61–7.66 (3H, m, H2˝, H4˝, H5˝) 7.81–7.90 (2H, m, H3’, H7’) 8.00 (1H, d, *J* = 4.6 Hz, H6’) 8.14 (1H, d, *J* = 4.7 Hz, H8’) 8.36 (1H, d, *J* = 4.6 Hz, H5’) 8.98 (1H, d, *J* = 4.6 Hz, H2’); ^13^C NMR (101 MHz, MeOD-d3) *δ*/ppm: 10.26 (C11) 20.66 (C10) 23.84 (C5) 23.95 (C7) 24.58 (C4) 35.44 (C3) 56.66 (C2) 56.91 (C6) 62.30 (CH_2_) 65.47 (C8) 67.96 (C9) 117.12 (C3’) 119.87 (C8’) 120.36 (C6’) 123.03 (C5˝) 124.75 (C9’) 127.80 (C7’) 128.85 (C6˝) 129.54 (C5’) 129.78 (C8˝) 129.78 (C8˝) 130.89 (C4˝) 145.97 (C1˝) 147.33 (C3˝) 149.65 (C2’) 161.61 (C10’) 164.06 (C4’); HRMS (Electrospray ionisation (ESI) m/z calcd for C_26_H_30_FN_2_O^+^ = 405.2337, found 405.2335 (mass error Δm = −0.49 ppm).

***N*-(4-fluorobenzyl)-10,11-dihydrocinchoninium bromide, DHCN 4F**. White solid. Yield: 26%; m.p. 271.0 °C; [𝛼] = +110 ° (c 0.1; MeOH); *t*_r_ 7.48 min; FTIR *ν̃*/cm^−1^: 3423 (O–H) 3136 (C–H) 1225 (C–N) 782 (C–F); **^1^H NMR** (400 MHz, MeOD-d3) *δ*/ppm: 0.95–0.98 (3H, t, *J* = 7.3 Hz, H11) 1.08–1.12 (1H, m, H5) 1.59–1.71 (2H, m, H10) 1.80–1.85 (3H, m, H4, H5, H7) 1.97 (1H, s, H7) 2.45 (1H, t, *J* = 11.8 Hz, H3) 3.04–3.12 (m, 1H, H2) 3.63 (1H, t, *J* = 11.1 Hz, H6) 3.85 (1H, m, H2) 3.98 (1H, t, *J* = 9.0 Hz, H6) 4.13–4.18 (1H, m, H8) 4.98 (1H, d, *J* = 12.5 Hz, H7˝) 5.08 (1H, d, *J* = 12.5 Hz, H7˝) 6.61 (1H, s, H9) 7.35 (2H, t, *J* = 8.5 Hz, H3’, H7’) 7.77–7.91 (4H, m, H2˝, H3˝, H5˝, H6˝) 8.00 (1H, d, *J* = 4.4 Hz, H6’) 8.15 (1H, d, *J* = 8.3 Hz, H5’) 8.33 (1H, d, *J* = 8.3 Hz, H8’) 8.98 (1H, d, *J* = 4.5 Hz, H2’); **^13^C NMR** (101 MHz, MeOD-d3) *δ*/ppm: 10.24 (C11) 20.64 (C10) 23.82 (C5) 23.92 (C7) 24.59 (C4) 35.40 (C3) 56.71 (C2) 56.78 (C6) 62.21 (C7˝) 65.48 (C8) 68.13 (C9) 119.86 (C3’) 122.99 (C6’) 124.75 (C9’) 127.06 (C5˝) 127.08 (C7’) 127.79 (C8˝) 128.88 (C8’) 129.80 (C6˝) 129.97 (C5’) 130.09 (C2˝) 131.06 (C1˝) 131.38 (C3˝) 137.36 (C4˝) 145.92 (C10’) 147.35 (C4’) 149.66 (C2’); HRMS (Electrospray ionisation (ESI) m/z calcd for C_26_H_30_FN_2_O^+^ = 405.2337, found 405.2338 (mass error Δm = 0.25 ppm).

***N*-(3-trifluoromethoxybenzyl)-10,11-dihydrocinchoninium bromide, DHCN 3OCF_3_**. White solid. Yield: 66%; m.p. 214.8 °C; [𝛼] = +90 ° (c 0.1; MeOH); *t*_r_ 8.16 min; FTIR *ν̃*/cm^−1^: 3441 (O–H) 3159 (C–H) 2954 (C–H) 1252 (-C–O–C-) 1161 (C–N) 782 (C–F); **^1^H NMR** (400 MHz, MeOD-d3) *δ*/ppm: 0.96 (3H, t, *J* = 7.4 Hz, H11) 1.12-1.08 (m, 1H, H5) 1.60–1.70 (2H, m, H10) 1.84–1.86 (3H, m, H4, H5, H7) 1.97 (1H, s, H7) 2.45 (1H, t, *J* = 11.8 Hz, H3) 3.05–3.12 (1H, m, H2) 3.64 (1H, t, *J* = 11.1 Hz, H6) 3.94–3.96 (1H, m, H2) 4.04 (1H, t, *J* = 9.3 Hz, H6) 4.18–4.22 (1H, m, H8) 5.06 (1H, d, *J* = 12.4 Hz, H7˝) 5.20 (1H, d, *J* = 12.4 Hz, H7˝) 6.62 (1H, s, H9) 7.56 (1H, d, *J* = 8.1 Hz, H7’) 7.72 (1H, t, *J* = 7.9 Hz, H3’) 7.79–7.88 (4H, m, H2˝, H4˝, H5˝, H6˝) 8.00 (1H, d, *J* = 4.5 Hz, H6’) 8.13 (1H, d, *J* = 8.0 Hz, H5’) 8.36 (1H, d, *J* = 8.0 Hz, H8’) 8.98 (1H, d, *J* = 4.5 Hz, H2’); ^13^C NMR (101 MHz, MeOD-d3) *δ*/ppm: 10.23 (C11) 20.66 (C10) 23.82 (C5) 23.97 (C7) 24.65 (C4) 35.40 (C3) 56.69 (C2) 56.83 (C6) 62.10 (C7˝) 65.47 (C8) 68.06 (C9) 119.24 (C1˝) 119.87 (C3’) 122.81 (C6’) 123.02 (C4˝) 124.75 (C9’) 126.10 (C5˝) 127.80 (C7’) 128.86 (C8’) 129.80 (C6˝) 129.95 (C8˝) 130.80 (C5’) 132.44 (C2˝) 145.93 (C10’) 147.33 (C4’) 149.39 (C3˝) 149.65 (C2’); HRMS (Electrospray ionisation (ESI) m/z calcd for C_27_H_30_F_3_N_2_O_2_^+^ = 471.2254, found 471.2257 (mass error Δm = 0.64 ppm).

***N*-(4-trifluoromethoxybenzyl)-10,11-dihydrocinchoninium bromide, DHCN 4OCF_3_**. White solid. Yield: 26%; m.p. 241.9 °C; [𝛼] = +77 ° (c 0.1; MeOH); *t*_r_ 8.43 min; FTIR *ν̃*/cm^−1^: 3435 (O–H) 3153 (C–H) 2883 (C–H) 1272 (-C–O–C-) 1161 (C–N) 785 (C–F); ^1^H NMR (400 MHz, MeOD-d3) *δ*/ppm: 0.96 (3H, t, *J* = 7.4 Hz, H11) 1.05–1.12 (m, 1H, H5) 1.59–1.73 (m, 2H, H10) 1.80–1.86 (m, 3H, H4, H5, H7) 1.97 (s, 1H, H7) 2.46 (t, 1H, *J* = 11.8 Hz, H3) 3.06–3.13 (m, 1H, H2) 3.62 (t, 1H, *J* = 11.0 Hz, H6) 3.92–3.97 (m, 1H, H2) 4.04 (t, 1H, *J* = 9.4 Hz, H6) 4.15–4.21 (m, 1H, H8) 5.02 (d, 1H, *J* = 12.5 Hz, H7˝) 5.16 (d, 1H, *J* = 12.5 Hz, H7˝) 6.63 (s, 1H, H9) 7.52(d, 2H, *J* = 8.0 Hz, H3’, H7’) 7.81–7.92 (m, 4H, H2˝, H3˝, H5˝, H6˝) 8.00 (d, 1H, *J* = 4.6 Hz, H6’) 8.14 (d, 1H, *J* = 4.6 Hz, H5’) 8.36 (1H, d, *J* = 7.9 Hz, H8’) 8.98 (1H, d, *J* = 4.6 Hz, H2’); ^13^C NMR (101 MHz, MeOD-d3) *δ*/ppm: 10.25 (C11) 20.65 (C10) 23.82 (C5) 23.96 (C7) 24.65 (C4) 35.40 (C3) 56.54 (C2) 56.80 (C6) 61.97 (C7˝) 65.48 (C8) 67.93 (C9) 119.18 (C1˝) 119.86 (C3’) 121.18 (C3˝. C5˝) 122.99 (C6’) 124.76 (C9’) 126.60 (C8˝) 127.79 (C7’) 128.87 (C8’) 129.79 (C5’) 135.58 (C2˝. C6˝) 145.95 (C10’) 150.69 (C4˝) 147.35 (C4’) 149.65 (C2’); HRMS (Electrospray ionisation (ESI) m/z calcd for C_27_H_30_F_3_N_2_O_2_^+^ = 471.2254, found 471.2257 (mass error Δm = 0.64 ppm).

### 3.3. Kinetic Measurements

#### 3.3.1. Enzymes

As a source of BChE, a purified human BChE, and as AChE, a recombinant human AChE was used, both enzymes kindly provided by Dr. F. Nachon (Département de Toxicologie, Armed Forces Biomedical Research Institute, France). The concentrated stocks of enzymes (BChE: 5.6 µM; AChE: 0.20 µM) were diluted in a phosphate sodium buffer 0.1 M (pH 7.4) containing 0.1% BSA before starting the experiments.

#### 3.3.2. Enzymes Activity Measurement

Enzyme activities were measured spectrophotometrically by the Ellman method [32] at 412 nM using 0.30 mM DTNB as thiol reagent, ATCh as substrate and water in 0.1 M phosphate buffer, pH 7.4. For the inhibition measurements, the reaction mixture contained tested compounds instead of water. No side interactions of tested compounds with ATCh or DTNB were detected. Measurements were done at 25 °C on a Tecan Infinite M200Pro plate reader (Austria). A detailed description of measurement protocol was described previously [18].

#### 3.3.3. Enzyme-Inhibitor Dissociation Constants

The reversible inhibition of BChE and AChE by tested compounds was measured by determining the decrease of enzyme activity towards ATCh in their presence. The activities of the enzymes were measured at different substrate concentrations ([S]; 0.050–0.50 mM) in the absence (*v*_0_) and presence (*v*_i_) of the given tested compounds concentration ([I]; 0.050–200 µM, depending on the compound) selected to inhibit the enzymes for 20–80%. At least three concentrations of inhibitors for each substrate concentration were used in at least three experiments. The apparent dissociation inhibition constant (*K*_i,*app*_) was calculated applying the Hunter-Downs equation and linear regression analysis (1):

(1) $$K_{i,app}=\frac{v_{i}}{v_{0}-v_{i}}\cdot \left[I\right]=K_{\left(I\right)}+\frac{K_{\left(I\right)}}{K_{\left(S\right)}}\cdot \left[S\right]$$

where *y*-intercept determines the enzyme-inhibitor dissociation constants (*K*_(I)_), while *x*-intercept determines the enzyme-substrate dissociation constant, *K*_(S)_. The determination of kinetic constants was carried out using the GraphPadPrism 6.0 program (GraphPad Software, San Diego, CA, USA).

### 3.4. In Silico Prediction of Drug-Likeness

The evaluation of compounds’ drug-likeness was carried out in terms of evaluation of their ability to pass through the intestinal after it is consumed orally and penetrate the lipid-based cell membrane from the blood. The drug-likeness evaluation was performed based on the values of physicochemical properties considered important for the ability of the compounds to be orally active drug in humans: molecular weight (MW), partition coefficient (logP), hydrogen bonds donors (HBD), hydrogen bond acceptors (HBA), number of rotatable bonds (RB) and polar surface area (PSA) [33,34]. The calculated values of those properties (Table 5) were compared to generally accepted recommended values: MW from 180 to 500, logP ranged from −0.4 to +5.6, HB ˂ 5, HBA ˂ 10 and RB ≤ 10, and PSA ˂ 140 Å**^2^** and those that have no more than one violation of those rules are orally active compounds [33,34,35]. The values of all physicochemical properties were calculated using the ChemAxon Chemicalize 2018 platform (accessed on 30 September 2022) [36].

### 3.5. In Silico Prediction of Blood-Brain Barrier Penetration

The ADMET descriptors protocol implemented in BIOVIA Discovery Studio 2018 predicts BBB penetration after the oral administration of a drug. The model contains a quantitative linear regression model for the prediction of blood-brain penetration, as well as 95% and 99% confidence ellipses derived from a correlation of the polar surface area (PSA-2D) and atom-based LogP (AlogP98) parameters derived from over 800 compounds known to enter the CNS after oral administration. Based on this model there are four BBB penetration levels: 0 (very high penetrant, logBBB ≥ 0.7), 1 (high penetrant, 0 ≤ logBBB < 0.7), 2 (medium penetrant, −0.52 < logBBB < 0) and 3 (low penetrant, logBBB ≤ −0.52). Log BBB denotes the base 10 logarithm of the ratio of the concentration of a compound measured in the brain to the concentration of the compound measured in the blood at a steady state [37].

### 3.6. Principal Component Analysis

Multivariate analyses were conducted by a 2nd-order tensor analysis tool known as principal component analysis (PCA) [38,39]. PCA data matrix ***X***, which has rank *r*, is decomposed in the sum of *r* matrices $t_{i}p_{i}^{\tau }$. Each matrix $t_{i}p_{i}^{\tau }$ has a rank of 1.

(2) $$X=\overset{r}{\underset{i=1}{\sum }}t_{i}p_{i}^{\tau }$$

$t_{i}$ is a vector of scores and $p_{i}^{\tau }$ is a vector of loadings. PCA provides the best linear projection of multidimensional data by minimizing the least squares objective function. Scores are used for classification, while loadings can be used for the variability identification among the data. PCA development goes back to Beltrami [40] and Pearson [41], and the name was introduced by Harold Hotelling [42].

Inhibition data were arranged in the data matrix ***X*** and PCA on the covariance matrix was performed by our parallelized code for multi- and univariate analysis [43,44,45]. Extraction of eigenvectors was based on the NIPALS algorithm [46] and obtained 1st principal component was subsequently used as a regressed variable.

### 3.7. Sampling of the Potential Energy Surfaces

*Ab initio* molecular dynamics simulations with *on-the-fly* calculations of forces were used as a sampling procedure for potential energy surfaces (PES). Equations were integrated using the velocity Verlet algorithm [47]. PM7 method [48] implemented in MOPAC2016 [49] was used for the calculation of forces at each point of the simulation. Molecular dynamics were conducted by using our *in-house* developed program *qcc* [50,51]. Phase space coverage was ensured by setting the initial temperature for Maxwell distribution of velocities to 773.15 K. During the simulation temperature was controlled using the velocity scaling algorithm. Step size was 0.5 fs and a total of 5,000,000 steps was computed for each compound. PES of compounds spanned in multidimensional space of Cartesian coordinates were decomposed by PCA providing principal components for further regression modelling.

### 3.8. Machine Learning Multivariate Linear Regression

Reduced spaces of measured inhibition data were used as dependent variables for estimation of fluorinated *Cinchona* alkaloids derivatives inhibition activities. Principal components were extracted by the 2nd-order tensor decomposition method. These principal components were regressed on the theoretically computed energy fingerprints of all compounds by performing extensive machine learning (ML).

ML procedure was applied for the generation of all possible multivariate linear regression models with a linear combination of original variables as well as their higher-order polynomial terms. Multivariate linear regression was performed using the following expression for matrices of coefficients B calculated by singular value decomposition:

(3) $$B={\left(X^{\tau }X\right)}^{-1}X^{\tau }Y$$

where X and Y are the matrices of independent and dependent variables, respectively. Every possible regression model of inhibition activity in dependence on molecular dynamics data was built and thoroughly validated by the *leave-one-out cross-validation* technique (LOO-CV). 3D models were inspected up to the 4^th^ order and the total number of investigated models was 17,179,869,184. The most optimal representations were selected based on the adjusted and predicted *R^2^* values, LOO-CV mean squared error as well as the total number of variables in the models.

## 4. Conclusions

We have prepared a series of 46 quaternized derivatives of cinchonidine and cinchonine as well as their 10,11-dihydro analogs. Quinuclidine nitrogen atom was quaternized with benzyl group having fluorine atoms in different positions, trifluoromethyl and trifluoromethoxy groups as well as combinations of them with bromine and chlorine atoms. Fluorination of compounds generally affected the acidity and basicity of the parent compound influencing compound’s binding affinity to cholinesterases, pharmacokinetic properties and bioavailability. All prepared compounds showed good to excellent inhibitory potential toward BChE with inhibition constants from nano- to micromolar range. CD derivative with a fluorine atom in *meta* position on the benzene ring was the most selective BChE inhibitor among the tested compounds showing 533 times higher preference for BChE. Also, based on in silico modelling all compounds possess blood-brain barrier penetration ability and inhibition potential toward enzyme CYP2D6. The best possible models of AChE and BChE inhibition with predicted *R*^2^(CD-derivatives) =0.9932 and *R*^2^(CN-derivatives) =0.9879 were calculated and cross-validated by utilizing an extensive machine learning protocol. Multivariate linear regression models with a linear combination of original variables and their higher-order polynomial terms were generated and tested. The best models among all generated and tested cases were determined. These activity/PES models can be used for accurate prediction of AChE and BChE inhibition for new similar compounds based solely on their potential energy surfaces calculated from ab initio molecular dynamics simulation enabling a smart guided search for new potential leads. These results strongly encourage further optimization of quaternized cinchonidine and cinchonine structural motifs in further research toward finding selective cholinesterase inhibitors.

## Figures and Tables

**Table 1:** Inhibition of AChE and BChE by derivatives of cinchonine and cinchonidine.

Compound	*K*_i_/µM		SI *	Compound	*K*_i_/µM		SI *
	BChE	AChE			BChE	AChE	
CD Bzl	0.075 ± 0.007	15 ± 2	200	CN Bzl	2.9 ± 0.3	121 ± 12	42
CD 2F	0.82 ± 0.03	33 ± 1	40	CN 2F	2.4 ± 0.1	80 ± 2	33
CD 3F	0.075 ± 0.005	40 ± 2	533	CN 3F	6.1 ± 0.3	13 ± 0.4	2.1
CD 4F	1.5 ± 0.1	69 ± 3	46	CN 4F	2.6 ± 0.1	3.9 ± 0.2	1.5
CD 3CF_3_	2.4 ± 0.1	34 ± 1	14	CN 3CF_3_	4.4 ± 0.2	59 ± 2	13
CD 4CF_3_	2.0 ± 0.1	21 ± 1	11	CN 4CF_3_	6.0 ± 0.3	31 ± 1	5.2
CD 3,5F	0.081 ± 0.01	10 ± 1	123	CN 3,5F	6.3 ± 0.2	34 ± 3	5.4
CD 3,4F	1.3 ± 0.1	13 ± 1	10	CN 3,4F	6.1 ± 0.2	14 ± 0.2	2.3
CD 2,3F	0.75 ±0.03	19 ± 1	25	CN 2,3F	9.6 ± 0.4	46 ± 3	4.8
CD 2,4F	6.1 ± 0.5	6.4 ± 0.3	1.1	CN 2,4F	6.0 ± 0.2	27 ± 1	4.5
CD 2,6F	9.9 ±0.4	7.7 ± 0.5	0.77	CN 2,6F	5.2 ± 0.2	30 ± 2	5.8
CD 3OCF_3_	7.4 ±0.4	8.2 ± 1.1	1.1	CN 3OCF_3_	4.7 ± 0.2	41 ± 2	8.7
CD 4OCF_3_	7.6 ± 0.5	7.3 ± 0.5	0.96	CN 4OCF_3_	7.8 ± 0.3	19 ± 2	2.4
CD 2F-6CF_3_	5.7 ± 0.6	35 ± 4	6.1	CN 2F-6CF_3_	7.7 ± 0.4	61 ± 2	7.9
CD 2F-4Br	0.68 ± 0.05	7.2 ± 0.4	10	CN 2F-4Br	5.5 ± 0.3	16 ± 1	2.9
CD 2Cl-6F	5.0 ± 0.3	9.9 ± 0.8	1.9	CN 2Cl-6F	1.2 ± 0.0	17 ± 1	14

* SI denotes stereoselective index calculated from *K*_i_(AChE)/*K*_i_(BChE).

**Table 2:** Inhibition of AChE and BChE by derivatives of 10, 11-dihydrocinchonine and 10, 11-dihydrocinchonidine.

Compound	*K*_i_/µM		SI *	Compound	*K*_i_/µM		SI *
	BChE	AChE			BChE	AChE	
DHCD	19 ± 2	206 ± 6	11	DHCN	1.2 ± 0.1	43 ± 2	43
DHCD Bzl	0.4 ± 0.02	4.8 ± 0.4	12	DHCN Bzl	0.9 ± 0.04	21 ± 1	23
DHCD 3F	0.3 ± 0.02	27 ± 2	84	DHCN 3F	1.2 ± 0.1	20 ± 1	20
DHCD 4F	4.3 ± 0.2	31 ± 1	8	DHCN 4F	1.6 ± 0.1	64 ± 2	40
DHCD 3CF_3_	1.4 ± 0.1	25 ± 1	18	DHCN 3CF_3_	1.2 ± 0.05	41 ± 2	34
DHCD 4CF_3_	3.2 ± 0.2	15 ± 1	5	DHCN 4CF_3_	1.6 ± 0.1	18 ± 1	9
DHCD 3OCF_3_	6.8 ± 0.3	36 ± 1	5	DHCN 3OCF_3_	1.3 ± 0.5	68 ± 1	52
DHCD 4OCF_3_	5.9 ± 0.2	17 ± 1	3	DHCN 4OCF_3_	2.2 ± 0.1	22 ± 1	10

* SI denotes selective index calculated as *K*_i_(AChE)/*K*_i_(BChE).

**Table 3:** Stereoselectivity of BChE and AChE toward *pseudo-enantiomers* of CN and CD.

		2F	3F	4F	3CF_3_	4CF_3_	3,5F	3,4F	2,3F	2,4F	2,6F	3OCF_3_	4OCF_3_	2F-6CF_3_	2F-4Br	6F-2Cl
***K_i_*(CN/CD)**	BChE	2.9	81	1.7	1.8	3.0	78	4.7	13	1.0	0.53	0.64	1.0	1.4	8.1	0.24
	AChE	2.4	0.33	0.056	1.7	1.5	3.4	1.1	2.4	4.2	3.9	5	2.6	1.7	2.2	1.7

**Table 4:** Stereoselectivity of BChE and AChE toward *pseudo-enantiomers* of 10, 11-dihydro compounds.

		-	Bzl	3F	4F	3CF_3_	4CF_3_	3OCF_3_	4OCF_3_
***K_i_*(DHCN/DHCD)**	BChE	0.063	2.2	4.0	0.37	0.86	0.50	0.19	0.37
	AChE	0.21	4.4	0.74	2.1	1.6	1.2	1.9	1.3

**Table 5:** Physicochemical properties (molecular weight (MW), partition coefficient (logP), hydrogen bonds donors (HBD), hydrogen bond acceptors (HBA), number of rotatable bonds (RB) and polar surface area (PSA)) of CD and DHCD compounds.

**Compounds**	MW/100	clogP	HBD	HBA	RB	PSA/10
CD 2F, 3F, 4F	4.83425	0.376	1	2	5	3.312
CD 3CF_3,_ 4CF_3_	5.33433	1.111	1	2	6	3.312
CD 3,5F, 3,4F, 2,3F, 2,4F, 2,6F	5.01416	0.519	1	2	5	3.312
CD 3OCF_3_, 4OCF_3_	5.49432	1.664	1	3	7	4.235
CD 2F-6CF_3_	5.51424	1.625	1	2	6	3.312
CD 2F-4Br	5.62321	1.145	1	2	5	3.312
CD 6F-2Cl	5.1787	0.98	1	2	5	3.312
DHCD	3.76319	2.975	1	3	3	3.636
DHCD Bzl	4.6616	0.537	1	2	5	3.312
DHCD 3F, 4F	4.85441	0.68	1	2	5	3.312
DHCD 3CF3, 4CF_3_	5.341493	1.415	1	2	6	3.312
DHCD 3OCF_3_, 4OCF_3_	5.550144	1.968	1	3	7	4.235
Recommended values	5	5	3	7	8	7

**Table 6:** In silico determined prediction of passive transport of compounds to the central nervous system (CNS) through the BBB, according to BIOVIA ADMET Protocol.

Penetration Levels *	0	1
**Compounds**	CD 3OCF_3_, CD 4OCF_3_, CN 3OCF_3_, DHCN 3OCF_3_, DHCN 4OCF_3_, DHCD 3OCF_3_, DHCD 4OCF_3_	CD 2F, CD 3F, CD 4F, CD 3CF_3_, CD 4CF_3_, CN 2F, CN 3F, CN 4F, CN 3CF_3_, CD 3,5F, CD 3,4F, CN 4CF_3_, CD 2,3F, CD 2,4F, CD 2,6F, CN 3,5F, CN 3,4F, CN 2,3F, CN 2,4F, CN 2,6F, CD 2F-6CF_3_, CD 2F-4Br, CD 2Cl-6F, CN 2F-6CF_3_, CN 2F-4Br, CN 2Cl-6F, DHCD, DHCD 3F, DHCD 4F, DHCD 4CF_3_, DHCD 3CF_3_, DHCD Bzl, DHCN, DHCN Bzl, DHCN 4CF_3_, DHCN 3F, DHCN 3CF_3_, Tacrine

* There are four BBB penetration levels: 0 (very high penetrant, logBBB ≥ 0.7), 1 (high penetrant, 0 ≤ logBBB < 0.7), 2 (medium penetrant, −0.52 < logBBB < 0) and 3 (low penetrant, logBBB ≤ −0.52).

## Data Availability

Data is contained within the article.
